# Allo-antibody production after intraarticular administration of mesenchymal stem cells (MSCs) in an equine osteoarthritis model: effect of repeated administration, MSC inflammatory stimulation, and equine leukocyte antigen (ELA) compatibility

**DOI:** 10.1186/s13287-020-1571-8

**Published:** 2020-02-07

**Authors:** Laura Barrachina, Alina Cequier, Antonio Romero, Arantza Vitoria, Pilar Zaragoza, Francisco José Vázquez, Clementina Rodellar

**Affiliations:** 1 Laboratorio de Genética Bioquímica LAGENBIO - Instituto de Investigación Sanitaria de Aragón (IIS), Universidad de Zaragoza, C/Miguel Servet, 177, 50013 Zaragoza, Spain; 2 Instituto Agroalimentario de Aragón (IA2), Universidad de Zaragoza – CITA, C/Miguel Servet, 177, 50013 Zaragoza, Spain; 3 Servicio de Cirugía y Medicina Equina, Hospital Veterinario, Universidad de Zaragoza, C/Miguel Servet, 177, 50013 Zaragoza, Spain

**Keywords:** Allogeneic, Immunogenicity, Joint, Horse, Humoral response, MSC priming, Major histocompatibility complex (MHC)

## Abstract

**Background:**

Antibody production after allogeneic administration of mesenchymal stem cells (MSCs) could impact their clinical application. Proinflammatory priming of MSCs can potentiate their regulatory ability in vivo but increased expression of major histocompatibility complex (MHC) might augment their immunogenicity, potentially leading to immune memory thus limiting repeated allogeneic administration. This study aimed at evaluating the production of cytotoxic allo-antibodies directed against donor’s ELA (equine leukocyte antigen) in mismatched and halfmatched horses receiving repeated intraarticular administration of stimulated MSCs (MSC-primed) and unstimulated MSCs (MSC-naïve) in pathologic joints.

**Methods:**

From available stored samples from a previous in vivo study, cells from one donor and serially collected sera (five time-points) from three groups of recipients were used based on their ELA haplotypes to perform microcytotoxicity assays: Group 1 recipients mismatched with the donor that received MSC-naïve (naïve-mismatched recipients); Group 2 recipients mismatched with the donor that received MSC-primed (primed-mismatched recipients); Group 3 recipients halfmatched with the donor (sharing 1/2 haplotypes) that received MSC-primed (primed-halfmatched recipients). Sera from recipients (neat, 1:2 and 1:16 dilution) were tested against target cells from the donor (cryopreserved and expanded MSC-naïve and MSC-primed) or from one animal presenting the same ELA haplotypes than the donor (fresh peripheral blood lymphocytes as control).

**Results:**

One to three weeks after first MSC administration, all recipient groups produced allo-antibodies regardless of MSC received (naïve or primed) and matching degree with donor. However, secondary response after MSC re-exposure was less evident in halfmatched recipients (MSC-primed) than in mismatched ones (both MSC-naïve and MSC-primed). Recipients of MSC-primed (both mismatched and halfmatched) tended towards developing lower antibody response than MSC-naïve recipients in vivo, but MSC-primed were targeted to death in higher percentage in vitro in the microcytoxicity assay.

**Conclusions:**

After first intraarticular allogeneic administration, the immunomodulatory profile of MSC-primed would have led to lower antibody production, but these antibodies would target more easily MSC-primed after second injection (re-exposure), likely because of their higher MHC expression.

**Electronic supplementary material:**

The online version of this article (10.1186/s13287-020-1571-8) contains supplementary material, which is available to authorized users.

## Background

The role of the horse as a sportive animal requires the proper function of its musculoskeletal system. Tendon, ligament or joint injuries represent about 80% of the causes of diminished performance with important direct and indirect economic loses [1]. Besides its importance as patient, the horse is considered one of the most suitable animal models of musculoskeletal pathologies, conferring to this species a huge importance for studying cell-based therapies for orthopedic diseases [2].

Therapeutic potential of mesenchymal stem cells (MSCs) is mainly attributed to their immunomodulatory properties, which are induced by exposure to an inflammatory environment [3]. Priming MSCs by proinflammatory cytokines in vitro may increase their regulatory effect in vivo [4]. However, this strategy may also raise MSC immunogenicity because of induced expression of major histocompatibility complex (MHC), thus potentially limiting the allogeneic administration [5] specially whether the development of immune memory mechanisms would limit repeated administration [6], which has been suggested to improve their therapeutic effectivity provided the limited effect of MSCs in the long term, especially the allogeneic ones [7].

Antibody production after allogeneic MSC administration has been reported in several species such as Rhesus Macaque [8], baboon [9], mouse [6], rat [10], pig [11, 12], and very recently, in the horse [13–15]. In rhesus macaques, the degree of MHC-mismatching between donor and recipient can affect the magnitude and type of the allo-immune response [8]. In horses, antibodies are specifically produced against the haplotype of the equine leukocyte antigen (ELA) of the donor when the recipient is ELA-mismatched, but not if donor and recipient are ELA-matched [13, 14]. This finding is of huge relevance, but clinical implications are yet unknown since these studies were conducted in healthy animals and the route of administration was intradermic, which is not relevant for clinical application. Another study detected antibody production in almost 40% of horses receiving allogeneic MSCs by different routes (intravenous, intraarterial, intratendinous and intraocular), but ELA haplotypes were not accounted [15]. Furthermore, full match or full mismatch has been assessed [13, 14], but semi-allogeneic scenario (halfmatched, i.e., one out of two haplotypes shared) was not evaluated and it could result in a more realistic scenario for clinical application [14].

In a previous study of our group, we assessed the repeated intraarticular (IA) administration of pooled allogeneic MSCs, both unstimulated (MSC-naïve) and primed with proinflammatory cytokines (MSC-primed) in a chemically induced (Amphotericin B IA injection) osteoarthritis (OA) equine model [16]. In this study, we observed beneficial effects of both treatments especially at the short term, but limited for the longer term. Furthermore, second MSC-primed administration led to a slight and transient local inflammatory reaction that was not observed after first injection, neither after any MSC-naïve administration [16]. These findings led us to hypothesize that, first, immune memory might have been developed thus limiting the duration of therapeutic effects, and second, that MSC-primed cells would be more intensively targeted because of their increased MHC expression [17]. Therefore, we conducted the present study aimed at assessing the production of allo-antibodies after allogeneic MSC administration by using stored samples from the previous study. The goal of the current study is to gain insight into humoral responses against allogeneic MSCs in a clinically relevant scenario that included an administration route not previously assessed (IA), pathologic condition (OA-model), repeated administration, use of both unstimulated and primed MSCs, and halfmatching for ELA haplotypes between donors and receptors.

## Methods

### Study design

**Fig. 1:**
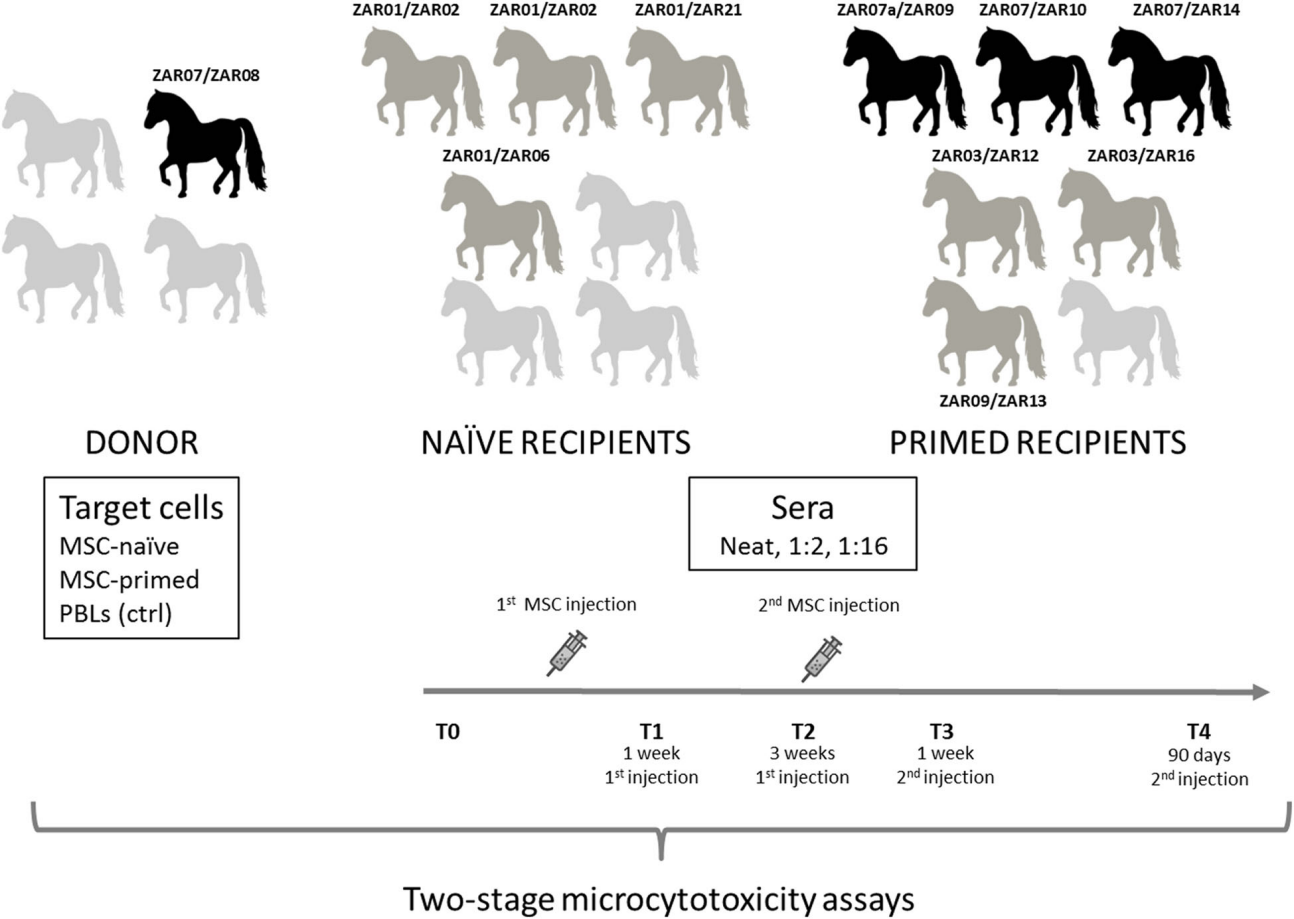
Schematic representation of the study design. From all the animals of the previous study, one donor (black), four recipients of MSC-naïve (all mismatched, dark gray), and six recipients of MSC-primed (three halfmatched, black; three mismatched, dark gray) were selected to assess humoral response against allogeneic mesenchymal stem cells (MSCs) based on their equine leukocyte antigen (ELA) haplotypes. Peripheral blood lymphocytes (PBLs), unstimulated MSCs (MSC-naïve), and MSCs pre-stimulated with tumor necrosis factor alpha and interferon gamma (MSC-primed) of the same ELA haplotype than the donor were used as target cells. Sera collected from the selected recipients at different time-points (T0, pre-administration of corresponding MSCs; T1, 1 week after first MSC administration; T2, 3 weeks after first MSC administration—just before the second MSC administration; T3, 1 week after second MSC administration; T4, 90 days after second MSC administration) were tested neat, 1:2 and 1:16 diluted against all the three types of target cells using two-stage microcytotoxicity assays

Samples came from a previous experiment in which all procedures were carried out under Project License (PI 31/11) approved by the Ethic Committee for Animal Experiments from the University of Zaragoza according to the Spanish Policy for Animal Protection RD53/2013, which meets the European Union Directive 2010/63. First, ELA haplotypes of donors (*n* = 4) and recipients (*n* = 7 recipients of MSC-naïve, *n* = 7 recipients of MSC-primed) of our previous study using IA administration of allogeneic MSCs were studied to assess the matching degree among them and select donor-recipient combinations, also based on sample availability: among the four donors, one was selected; among the seven MSC-naïve recipients, four were selected (all mismatched with the donor tested: group naïve-mismatched recipients); among the seven MSC-primed recipients, six were selected (three halfmatched with the donor: group primed-halfmatched recipients; and three mismatched with donor: group primed-mismatched recipients). Second, two-stage microcytotoxicity assays were carried out by mixing serially collected sera from selected recipients (potentially containing antibodies anti-ELA; neat, 1:2 and 1:16 diluted) and target cells with the ELA haplotypes of the donor selected (peripheral blood lymphocytes [PBLs], MSC-naïve, MSC-primed; whose expression of MHC types I and II was assessed by flow cytometry). Subsequently, rabbit complement was added and eosin Y dye exclusion was used to determine cell death percentage. Time-points selected were as follows: T0 (pre-administration of MSCs), T1 (1 week after first MSC administration), T2 (3 weeks after first MSC administration; just before the second MSC administration), T3 (1 week after second MSC administration) and T4 (90 days after second MSC administration). Variables of the study are summarized in Table 1, and the study design is outlined in Fig. 1.

**Table 1:** Summary and description of the variables of the study

Donor		Recipients			
ELA haplotypes	Target cells	Group	ELA haplotypes	Time-points of sera harvesting	Sera dilution
ZAR07/ZAR08	PBLs^1^ (control) MSC-naïve (unstimulated) MSC-primed (5 ng/ml TNFα and 5 ng/ml IFNγ for 12 h)	Naïve-mismatched: • Injected with MSC-naïve • ELA-mismatched with donor (0/2 haplotypes shared) • *N = 4*	ZAR01/ZAR21 ZAR01/ZAR02 ZAR01/ZAR02 ZAR01/ZAR06	T0 = pre-administration of MSCs T1 = 1 week after 1st MSC administration T2 = 3 weeks after 1st MSC administration (just before 2nd administration) T3 = 1 week after 2nd MSC administration T4 = 90 days after 2nd MSC administration	Neat (no dilution) Dilution 1:2 Dilution 1:16
		Primed-halfmatched: • Injected with MSC-primed • ELA-halfmatched with donor (1/2 haplotypes shared) • *N = 3*	ZAR07a/ZAR09 ZAR07/ZAR10 ZAR07/ZAR14		
		Primed-mismatched: • Injected with MSC-primed • ELA-mismatched with donor (0/2 haplotypes shared) • *N = 3*	ZAR03/ZAR12 ZAR03/ZAR16 ZAR09/ZAR13		

ELA haplotypes of the donor selected and type of target cells; groups of recipients according to MSCs received and their haplotypes (when possible, sharing of one haplotype among recipients was used as criteria to select more homogeneous groups: naïve-mismatched recipients all shared the haplotype ZAR01; primed-halfmatched recipients shared the haplotype ZAR07 among them and with the donor - one of the recipients presented the variation ZAR07a; two primed-mismatched recipients shared the haplotype ZAR03 and the other one did not share any haplotype); time-points at which sera was collected from recipients and sera dilutions assessed. *MSCs* mesenchymal stem cells, *ELA* equine leukocyte antigen, *TNFα* tumor necrosis factor alpha, *IFNɣ* interferon gamma, *T* time, *PBL* peripheral blood lymphocyte. ^1^PBLs were obtained from a different horse but with the same ELA haplotypes than the donor selected

### Determination of ELA haplotypes

Genomic DNA was extracted from frozen samples (− 80°) of synovial fluid (SF) of all the 18 animals enrolled in the previous study (Shetland ponies, geldings, 3–7 years, 100–165 kg) using the Quick-gDNA™ Miniprep Kit (Zymo Research) according to the manufacturer’s instructions. Horses were genotyped using a panel of 10 highly polymorphic intra-MHC microsatellites previously validated [18] that included the following markers: COR110, COR112, COR113, COR114, UM011 [19], UMNJH-38, ABGe9019, UMNe65, ABGe9030, and EQMHC1 [20]. Dr. Antzack and Dr. Miller (Cornell University) kindly provided DNA samples of known haplotypes analyzed in their laboratory to be used as reference samples to correctly assign fragment lengths. Fluorescently labeled primers were purchased from Invitrogen using the sequences previously published (Table 2).

**Table 2:** Primers used for amplification of horse intra-MHC microsatellites

	Locus		Dye	Primer sequence	Allele range (bp)	Reference
MHC Class I	UMNJH-38	F′	FAM	TGTGTGTGCACCTGTCCTTT	156–165	Sadeghi et al., 2018 [18]
		R′		GATGGGAGGGAATGAGGAAT		
	COR110	F′		TTTGGTCTTTGCAGGTATGG	194–221	Tseng et al., 2010 [19]
		R′	VIC	TCTCCCTTCCTCTTTGTTCC		
MHC Class III	ABGe 9019	F′	FAM	CTGAGAGAGACAGCATTTGTGG	297–320	Sadeghi et al., 2018 [18]
		R′		GAAAGGTGTCTCCATTCTTGCT		
	UNMe65	F′	AT550 (NED)	TCGCAAAACCCACAGACTAC	247–269	Sadeghi et al., 2018 [18]
		R′		TTCTCCTTTCCTTCCACTCC		
MHC Class II	ABGe 9030	F′	AT565 (PET)	CCAGCAGACCTGCAAGAGTA	205–221	Sadeghi et al., 2018 [18]
		R′		AGCATGAGAGCCATGAAGGT		
	EQMHC1	F′	AT532 (VIC)	ATGCATACCGGGAAAGACAG	180–196	Sadeghi et al., 2018 [18]
		R′		AGAGACTTCAGTCTCTGTGGTG		
	COR112	F′		TTACCTGGTTATTGGTTATTTGG	236–268	Tseng et al., 2010 [19]
		R′	NED	TCACCCACTAAATCTCAAATCC		
	COR113	F′		TGTTTAGAACTCGCCAGGAG	260–276	Tseng et al., 2010 [19]
		R′	FAM	TCATCAGTTCCTTGCCTAGC		
	UM011	F′		TGAAAGTAGAAAGGGATGTGG	165–180	Tseng et al., 2010 [19]
		R′	FAM	TCTCAGAGCAGAAGTCCCTG		
	COR114	F′		TCAAAATCCACACTCCCTTC	234–255	Tseng et al., 2010 [19]
		R’	PET	TCCATAAAGAGTGGGACACTG		

Primers (F′, forward; R′, reverse), dye, sequence, allele range (base pair) and reference

Two multiplex polymerase chain reactions (PCR) were used for amplification: PCR 1 included COR110, COR112, COR114, and UM011 and PCR 2 included UMNJH-38, ABGe9019, UMNe65, ABGe9030, and EQMHC1. The marker COR113 was separately assayed. Each PCR mix contained 3 μL of 2× PCR Master-mix, 0.6 μL 5× Q-solution, 0.5 μL of primers mix (equal volumes of each one) and 0.9 μL RNAse-free water (Qiagen Multiplex PCR Kit, Qiagen) making a total volume of 5 μL per sample; 1 μL DNA was used for each reaction. Amplification protocol consisted of one cycle of 95 °C 15′, followed by 30 cycles of 95 °C 30″, 58 °C 1′ and 72 °C 1′, and one cycle of 72 °C 30′ for final extension. Fragment analysis was performed by mixing 2 μL of PCR product (diluted 1:5 in water) with 12 μL Hi-Di™ formamide (Applied Biosystems) and 0.3 μL GeneScan™-500 LIZ™ Size Standard (Applied Biosystems). Electrophoresis of the samples was carried out on an ABI 3130 Genetic Analyzer (Applied Biosystems), and fragment lengths were analyzed with the GeneMapper® v3.7 software (Applied Biosystems), using reference samples as aforementioned.

### Rationale for selection of donor-recipient combinations

To perform microcytotoxicity assays, three types of target cells were used: PBLs, MSC-naïve and MSC-primed. Since high viability of target cells for microcytotoxicity assays is required [13], cryopreserved MSCs from the selected donor were thawed and expanded to ensure high viability of the cells. However, cryopreserved PBLs were not available and, furthermore, their viability after freezing might have been compromised [21] so fresh PBLs were needed. Provided donors of our previous study were enrolled as control group in that study, these animals were also euthanized at the end of the study so it was not possible to obtain fresh blood from the exact same donors. Therefore, other animals from the livestock of the donors were analyzed to find animals with the same ELA haplotypes. One of the tested animals presented the two same ELA haplotypes than one of the donors, so fresh PBLs were obtained from this animal under signed owner’s consent. Because of this limitation, this was the only donor that we could assess.

Based on the ELA haplotypes of the selected donor, recipients were chosen following two criteria: first, matching degree regarding donor; second, matching degree among recipients within the same group. This approach was established to take into account the relationship between donor and recipients (fully or partially mismatched) and to try to establish recipient groups as homogeneous as possible (preferably sharing at least one haplotype within the group). Haplotypes assigned to the animals selected for this study are presented in Table 1 to clarify the rational choice and grouping of animals.

### Preparation of target cells for microcytotoxicity assays

Peripheral blood lymphocytes were isolated using the carbonyl iron granulocyte depletion method followed by density gradient centrifugation with Ficoll as previously described [13]. Briefly, blood was collected via jugular venipuncture into sterile blood collection tubes with 170 units of lithium heparin (BD) and plasma was allowed to separate for 20′ at room temperature (RT). Plasma was collected and incubated with carbonyl iron (Sigma-Aldrich) in agitation for 30′ at 37 °C. Then, carbonyl iron was placed in the bottom of the tubes by using a magnet, and supernatant was collected and centrifuged 310*g* 10′. The cellular pellet was resuspended in PBS (Gibco) and overlayed on Lymphoprep (Atom). After 690*g* 15′ centrifugation, cell lay was recovered and washed with PBS. This isolation technique has been reported to provide enriched lymphocyte population (95–99%) and has been widely used in related research [13, 14, 22]. Cells were counted in a hemocytometer chamber and viability was calculated by using Trypan Blue 0.4% dye exclusion. Concentration was adjusted to 3 × 10^6^ live cells/mL to be used immediately afterwards for microcytotoxicity assays.

Frozen BM-MSCs from selected donor at passage 3 were thawed and expanded at 37 °C 5% CO_2_ in culture medium consisting of low glucose Dulbecco’s modified Eagle’s medium (DMEM) supplemented with 10% fetal bovine serum (FBS), 2 mM l-glutamine, 0.1 mg/mL streptomycin and 100 U/mL penicillin (all from Sigma-Aldrich). Cells from this donor were characterized as MSCs in a previous study [17]. Four days before performing microcytotoxicity assays, serum component of the medium was changed by 5% FBS and 5% equine serum of the same ELA haplotype than the MSCs (i.e., from PBLs’ donor). After 48 h, medium was replaced by a new one containing only 10% equine serum during additional 48 h. To obtain MSC-primed, BM-MSCs were exposed to 5 ng/mL of TNFα and 5 ng/mL of IFNγ (both from R&D Systems) (added to fresh medium also containing 10% equine serum) during 12 h [16] before conducting microcytotoxicity. Naïve (unstimulated) and primed BM-MSCs were detached from plastic with 0.25% trypsin-EDTA (Sigma-Aldrich) and washed in PBS. Counting and viability calculation were performed as aforementioned for PBLs. Viability of all the three types of target cells (PBLs, MSC-naïve, and MSC-primed) was > 94%. Previous reports of our group showed appropriate viability of MSCs after inflammatory priming under these conditions [17]. Cell concentration was adjusted to 1 × 10^6^ cells/mL in PBS to assay microcytotoxicity immediately after. An aliquot of both MSC-naïve and MSC-primed was used for flow cytometric analysis.

### MHC-I and MHC-II analysis by flow cytometry

Surface expression of MHC-I and MHC-II was studied by flow cytometry in both MSC-naïve and MSC-primed since inflammatory exposure can induce changes in MHC expression. Methodology followed and antibody suitability was previously described [23]. Briefly, the cells were suspended in PBS/2 mM EDTA at 10^6^ cells/ml and aliquots of 50 μl were transferred to FACS tubes and incubated 15′ at 4 °C in the dark with monoclonal antibodies anti-HLA-ABC-FITC (Beckman Coulter) and HLA-DR-APC (Immunostep). Subsequently, cells were washed with PBS and diluted in 500 μl of PBS/2 mM EDTA for analysis in the flow cytometer (FACSARIA, BD Biosciences). Sytox Blue was used to gate live cells. Data were analyzed with FACSDIVA 5.0.1 software.

### Two-stage microcytotoxicity assays

The standard two-stage microcytotoxicity dye exclusion assay was carried out to detect cytotoxic antibodies as previously described [13, 14] using MSC-naïve and MSC-primed as target cells in addition to PBLs. The three types of target cells were assayed against sera (neat, 1:2 and 1:16 diluted) from a total of 10 recipients and 5 time-points as stated in the Study Design. Serum of the same ELA haplotype than MSC donor (i.e., from PBL donor) was used neat as negative control. As positive control, serum from one of the animals in the previous study that was not included in the current one was used. To select this positive control, sera from the two animals that showed stronger local reaction after MSC-primed administration (collected 1 week after second injection) were tested against PBLs and one antisera was selected based on providing > 80% of cell death. Frozen sera from the previous in vivo study were thawed, centrifuged 12,000*g* 10′ at 4 °C, and diluted in natural horse serum (Pan-Biotech). One microliter of corresponding serum was incubated with 1 μL of corresponding target cell suspension in Terasaki plates (One Lambda) under mineral oil (Sigma-Aldrich) during 30′ at RT. Subsequently, 5 μL of rabbit complement (One Lambda) was added and plates were incubated for 1 h RT. Then, 2 μL of 5% Eosin Y dye (Sigma-Aldrich) were added to each well and after 5′ cells were fixed with 5 μL/well of 10% formalin (Sigma-Aldrich, pH 7.2–7.4). Two technical replicates were performed for each condition (type of target cell, recipient, time, and serum dilution) and controls (positive and negative). The plates were refrigerated and the percentage of dead cells was assessed within 24 h by the two first authors (LB and AC) using a phase-contrast (× 10 magnification) Leica inverted microscope (live cells are refringent and rounded; dead cells are dark, flat and irregular) and following the scoring system described by Berglund and Schnabel [13] (score 1 < 10%, score 2 10–19%, score 4 20–49%, score 6 50–80%, score 8 81–100%). According to these authors, scores of 6 or higher (> 50% cell death) and scores of 2 or lower (< 20% cell death) in neat serum were considered to reflect significant and non-significant presence of allo-antibodies in sera, respectively. The average cytotoxicity score was calculated for each condition and used for statistical analysis.

### Statistical analysis

Statistical analysis was carried out with the SPSS 20.0 software (SPSS, Inc.). Interrater agreement was calculated with Cronbach’s alpha for each type of target cells (PBLs, *α* = 0.966; MSC-naïve, *α* = 0.970; MSC-primed, *α* = 0.944), and thus, mean scores were calculated and used for subsequent statistical analysis. Descriptive analysis was performed by calculating the overall percentage of animals with scores in neat sera over 6 (considered as significant antibody production) and below 2 (considered as non-significant antibody production) within each recipient group. Analytical statistics were performed to check differences in scores depending on the different study variables. The variables were “type of receptor” (three categories: naïve-mismatched, primed-mismatched, primed-halfmatched), “type of target cells” (three categories: PBLs, MSC-naïve, MSC-primed), “time-point” (five categories: T0, T1, T2, T3, T4) and “score” (three categories: score for neat sera, score for 1:2 diluted sera, score for 1:16 diluted sera). “Score” was set as the dependent variable and each other variable as factor to study differences as follows: (1) differences along time within each group of recipients for each type of target cells, (2) differences among types of target cells at each time-point within each group of recipients, (3) differences among recipient groups for each type of target cell at each time-point. One-way ANOVA was performed with Bonferroni or Games-Howell as post hoc, assessing the homogeneity of variances by Levene’s test. Significance level was set at *p* < 0.05 for all analysis.

## Results

### Assignation of ELA haplotypes

All of the 18 animals enrolled in the previous in vivo study were heterozygotes for ELA haplotype. Since familiar relationship among animals was unknown and none of the animals was homozygote for all the markers, haplotypes could only be assigned provisionally. A total of new 22 provisional haplotypes were found in 17 animals involved in the previous in vivo study, whereas one animal could not be assigned with any haplotype. None of the haplotypes observed in this population was previously described [18, 24]. Further detailed information about ELA microsatellite typing is available in Additional file 1.

### MHC-I and MHC-II surface expression

Percentage of MHC-I positive cells was 0.9% for MSC-naïve and 74.2% for MSC-primed. For MHC-II, 0.2% of MSC-naïve were positive whereas 24.4% of MSC-primed expressed MHC-II. These results corroborated the increased expression of both MHC type I and II in MSC-primed used as target cells in microcytotoxicity assays.

### Overall descriptive analysis of allo-antibody production

**Fig. 2:**
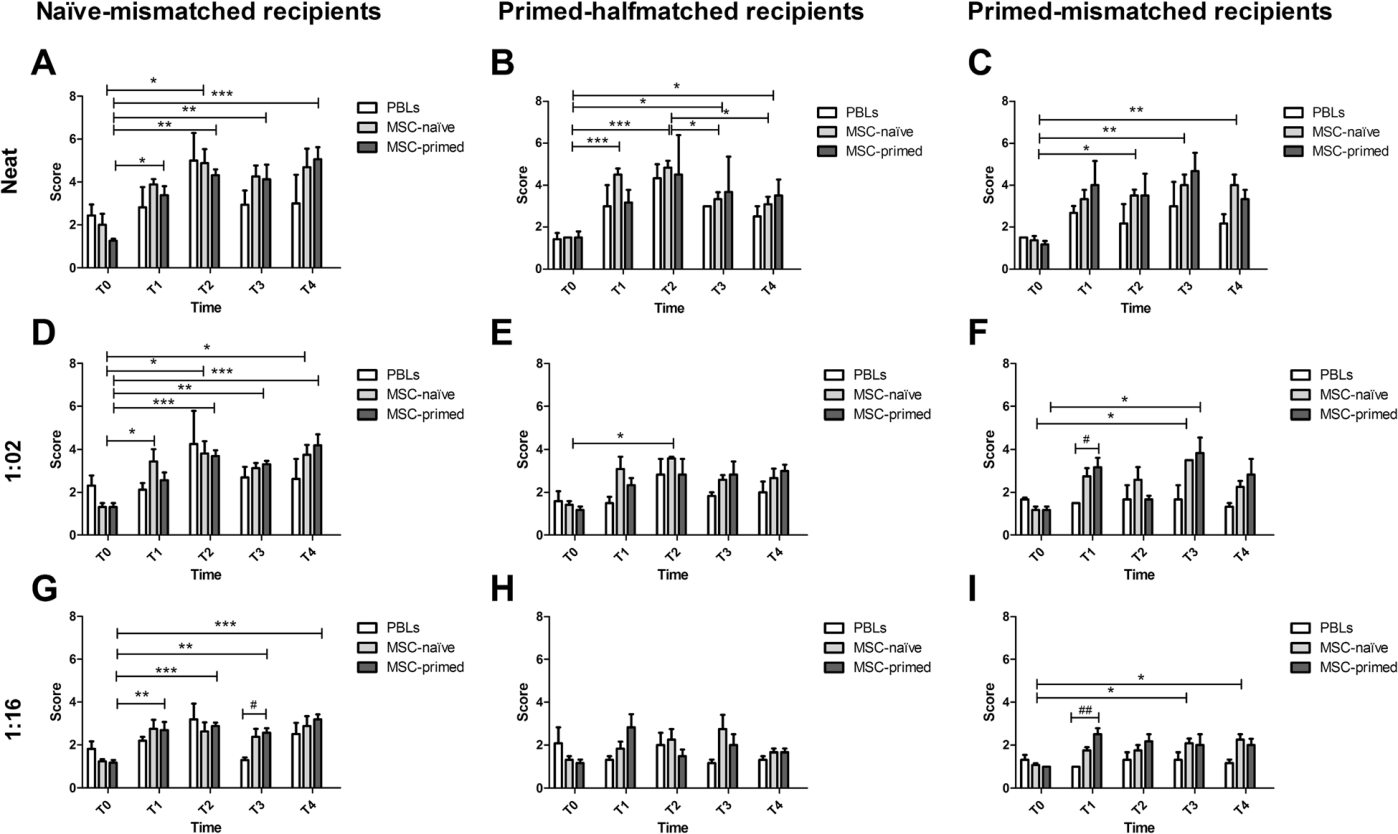
Evolution of cytotoxic scores along the time in each recipient group comparing different types of target cells. Mean ± S.E.M. of cytotoxic scores (*Y* axis) assigned to neat sera (top row; **a**, **b**, **c**), 1:2 diluted sera (middle row; **d**, **e**, **f**), and 1:16 diluted sera (bottom row; **g**, **h**, **i**) from mismatched recipients of MSC-naïve (left column; **a**, **d**, **g**) and MSC-primed, halfmatched (middle column; **b**, **e**, **h**) and mismatched (right column; **c**, **f**, **i**), along the time (*X* axis; T0, pre-administration of corresponding MSCs; T1, 1 week after first MSC administration; T2, 3 weeks after first MSC administration—just before the second MSC administration; T3, 1 week after second MSC administration; T4, 90 days after second MSC administration), when assayed against different target cells: PBLs, peripheral blood lymphocytes (white bar, control); MSC-naïve, unstimulated mesenchymal stem cells (light gray bar); MSC-primed, mesenchymal stem cells pre-stimulated with tumor necrosis factor alpha and interferon gamma (dark gray bar). Asterisks (*) point out statistically significant differences among time-points (* = *p* < 0.05, ** = *p* < 0.01, *** = *p* < 0.001) and hashes (#) indicate significant differences between different target cells at one particular time (# = *p* < 0.05, ## = *p* < 0.01)

After IA administration of allogeneic MSCs—naïve or primed—regardless of the ELA matching degree (halfmatched or mismatched), cytotoxic antibodies were produced. Mean scores in neat sera were below 6 in all the conditions (recipient group, type of target cell and time) (Fig. 2a–c). Pre-existing antibodies were not detected; all samples were scored as < 2 at T0 except for two recipients of MSC-naïve, which were assigned > 2 but < 6. These animals might have had pre-existing antibodies, for example from a previous transfusion, since their whole previous medical record is unknown. However, we decided to maintain these animals in the study design since their sera scores kept below 6 at all the posterior times, thus considering that an accelerated humoral response by previous exposure was unlikely.

Taking into account all the time-points and considering overall the different combinations of neat sera and target cells, 18.75% of sera samples from naïve-mismatched recipients, 5.6% from primed-halfmatched, and 5.6% from primed-mismatched groups showed a score > 6. On the other hand, the percentage of sera samples scored as < 2 was 12.5% for naïve recipients, 13.9% for primed-halfmatched recipients, and 22.2% for primed-mismatched recipients.

### Allo-antibody production along the time: primary and secondary humoral response

Along all the time-points assessed, changes in sera scores when incubated with PBLs were not statistically significant, neither neat or at any dilution. These scores followed the same trend when sera were incubated with MSCs—naïve or primed—as target cells, but observing significant changes along the time (Fig. 2). For the sake of clarity, in this section, time evolution will be presented for each recipient group regardless of the type of MSCs targeted, and differences between target cells will be presented in the next section. Nevertheless, it is worth mentioning that in naïve-mismatched recipients most of the significant differences along time were found when using MSC-primed as target cells. However, the opposite was observed in both mismatched and halfmatched MSC-primed recipients, in which most of the significant changes were observed when using MSC-naïve in the microcytotoxicity assays.

Scores from neat sera in the three recipient groups significantly increased over T0 at T2, T3, and T4, and in naïve-mismatched and primed-halfmatched recipients also at T1 (Fig. 2a–c). In naïve-mismatched (*p* < 0.01, Fig. 2a) and primed-halfmatched (*p* < 0.001, Fig. 2b) recipients, mean scores peaked at T2 (3 weeks after first injection), whereas in primed-mismatched recipients maximum mean values were observed at T1 despite no statistical significance (Fig. 2c). One week after second injection (T3), scores in naïve-mismatched and primed-halfmatched recipients diminished, only significantly over the previous time-point (T2) in primed-halfmatched recipients (*p* < 0.05, Fig. 2b). On the other hand, primed-mismatched recipients increased mean scores at T3 (*p* < 0.01, Fig. 2c). At T4 (90 days after second injection), scores increased again in naïve-mismatched recipients (*p* < 0.001 over T0, Fig. 2a). However, in primed-halfmatched recipients, scores at T4 remained higher than at T0 (*p* < 0.05) but significantly decreased over T2 (*p* < 0.05) (Fig. 2b), being the only group that significantly diminished scores after MSC re-exposure. Scores at T4 in primed-mismatched recipients diminished but were maintained significantly higher over T0 (*p* < 0.01) (Fig. 2c).

Serial dilutions of sera followed the same trend in the three groups of recipients with lower mean scores. Scores in 1:2 and 1:16 diluted sera from naïve-mismatched recipients were significantly increased over T0 along all time-points (Fig. 2d, g), as well as with neat sera. On the other hand, only few significant differences were observed in diluted sera from recipients of MSC-primed. In primed-halfmatched recipients, scores at T2 significantly increased over T0 in 1:2 diluted (*p* < 0.5) (Fig. 2e) and no significant differences along time were observed for 1:16 diluted sera (Fig. 2h). In the primed-mismatched group, significant increase over T0 was found at T3 in 1:2 (*p* < 0.05) and 1:16 (*p* < 0.05) diluted sera (Fig. 2f), plus at T4 in 1:16 dilution (*p* < 0.05) (Fig. 2i).

Summarizing, both primary and secondary humoral responses were detected in mismatched recipients of MSC-naïve and MSC-primed, the second ones tending towards a faster development of antibody production. On the other hand, secondary response was not apparent in halfmatched recipients of MSC-primed and even a significant decrease in cytotoxic scores was noticed after re-exposure.

### Cytotoxicity of allo-antibodies on different target cells

When analyzing cytotoxicity against the different types of target cells, it was observed a trend towards higher percentage of cell death in MSC-primed compared to PBLs, finding some punctual significant differences only in 1:2 and 1:16 diluted sera but not in neat sera. Specifically, MSC-primed were significantly more targeted than PBLs when exposed to sera (dilution 1:16) from naïve-mismatched recipients at T3 (Fig. 2g) and from primed-mismatched recipients at T1 (dilution 1:2, *p* < 0.05, Fig. 2f; dilution 1:16, *p* < 0.01, Fig. 2i).

### Allo-antibody production among recipients: effect of MHC matching (mismatching or halfmatching) and type of MSCs received (naïve or primed)

When comparing cytotoxic scores between the three recipient groups at each time-point, an overall trend towards lower scores in sera from MSC-primed recipients (both halfmatched and mismatched) was found. Statistically significant differences were only punctually observed for different target cells, times, and sera dilution (Additional file 2).

## Discussion

The results of this study corroborate the production of allo-antibodies with cytotoxic capacity directed against the MHC of administered allogeneic MSCs. This is the first study in the equine species reporting humoral response against allogeneic MSCs repeatedly IA administered in OA joints and also taking into account partial MHC compatibility and the role of proinflammatory MSC priming.

Nevertheless, this study presents some limitations that must be considered before discussing the results. First, halfmatched recipients of MSC-naïve could not be assessed neither fully matched donor-recipients could be used as control, since the matching degree between donors and recipients was not set up in the original study. Nevertheless, previous studies have reported the lack of immune response when donor and recipient are ELA-matched, both in vitro [22] and in vivo [13, 14]. Second, ELA haplotypes could only be provisionally assigned due to the absence of homozygous or family-related animals in the study. Third, MSCs from four donors were administered as a pool in the previous in vivo study [16] but allo-antibody production could only be studied against ELA haplotypes of one of the donors, yet cross-reactivity of anti-ELA antibodies cannot be excluded [14].

In this study, antibody peak production was observed between 1 and 3 weeks after first administration of allogeneic MSCs. Similarly, the first study reporting allo-antibody production against MHC-mismatched MSCs in the equine species [14] showed a peak in antibody production between 2 and 3 weeks after administration in 4 out of the 6 horses assessed. In the remaining two recipients, humoral response was weak so a second administration was performed. In one of them, slight response was observed whereas the other horse did not respond to re-exposure. Therefore, considerable variability could be expected between recipients in terms of humoral response against allo-MSCs [14]. A continuation of that study tested the sera from the four reactive horses with MSCs as target cells, showing that microcytotoxicity against these cells also increased from 1 to 3 weeks post-administration [13].

In the aforementioned studies, effect of re-injection in animals already showing a primary humoral response was not tested and only intradermal route was assessed. There is a third study on antibodies against equine allo-MSCs that assessed different routes of administration and repeated doses of allogeneic cells of unknown ELA [15]. This study found that almost 40% of horses that received allogeneic MSCs by different routes developed a humoral response, with more marked response in animals injected intratendinous, which also received higher number of administrations (five injections). However, in this study, the specificity of the antibodies against MHC was not tested and only the end-point was assessed, but not the evolution along the time of the humoral response and the influence of each administration. Therefore, studies on the evolution of humoral response along the time and the effect of repeated administration are needed in horses.

In our study, evolution of cytotoxic scores along the time in sera from different recipients suggested the development of both primary and secondary (after first and second MSC injection, respectively) humoral responses. This dynamic was observed in mismatched recipients of both MSC-naïve and MSC-primed, the second ones apparently showing faster development of antibody production. However, secondary response was not apparent in halfmatched recipients after second injection of MSC-primed. It could be hypothesized that when recipients of MSC-primed were partially matched with the donor, primary humoral response would be produced similarly to mismatched recipients of MSC-naïve, but secondary humoral response would be attenuated after re-exposure. It should also be considered that this apparent lack of secondary response may also be due to rapid elimination of MSC-primed after second injection by already existing antibodies, having no time to produce a secondary response [14]. However, animals that also received MSC-primed but were completely mismatched with the donor did develop a secondary humoral response, suggesting that MSC-primed would not be so rapidly removed.

Only few studies in other species have also assessed the effect on the humoral response of priming MSCs with IFNγ, finding no differences regarding antibody production compared to unstimulated ones [10], or finding them only after multiple injection of allogeneic primed MSCs [12]. We hypothesize that increased immunomodulatory activity of MSC-primed [17] together with partial matching for MHC might have attenuated the development of a secondary humoral response, being these factors potentially beneficial for allogeneic therapy. On the other hand, overall trend observed in mismatched recipients of MSC-primed suggested faster development of both primary and secondary humoral responses than in the other groups. Faster humoral responses are associated to previous exposure [11, 25], but pre-existing antibodies were not found in any animal of this group. Therefore, it might be hypothesized that higher MHC expression of MSC-primed would accelerate the humoral response in mismatched recipients, whereas it might be attenuated in halfmatched ones.

The fact that significant differences along the time were found in sera from naïve recipients mostly against MSC-primed as target cells, and vice versa in both groups of MSC-primed recipients, is an unexpected observation, but we were unable to attribute a biological meaning. It is possible that it could be due just to the lower deviation in scores recorded when using one or another type of target cell in each group.

Lack of humoral response in animals that received ELA-matched allogeneic MSCs (2/2 haplotypes shared) has been previously described [13, 14], but the effect of partial matching (1/2 haplotypes shared) on antibody development was not previously reported in the horse. In rhesus macaques [8] and humans [26], partial MHC matching has been accounted as sharing of some alleles—but not of complete haplotype—between donor and recipient, finding a protective effect of partial matching in rhesus macaques, and no significant effect in humans. Halfmatching represents a more realistic clinical scenario, provided that in practice it is difficult to find donors completely matched with the patient because of the great variability of ELA haplotypes [18, 19] that was also observed in this study even with limited population. According to our data, partial MHC matching would not prevent antibody production but might have attenuated the secondary response after re-exposure.

In this study, average scores tended to be lower than those previously reported, with less than 11% of all samples scored above 6. Overall, cytotoxic scores were below the limit setup for significant response both after first and second administration of either MSC-naïve or MSC-primed. Therefore, accordingly to previous reports in human medicine, the clinical relevance of this immune reaction for single injection may not be a major concern [26, 27]. However, this primary humoral response could compromise the effectiveness and safety of repeated administration as transient but significant increase in some inflammatory parameters were detected in the in vivo study after the second injection of MSC-primed [16]. Accordingly, a recent study assessing allo-sensitization after single administration of allogeneic MSCs in Chron’s disease patients showed generation of donor-specific antibodies (DSA) just in a proportion of patients, but those presenting pre-existing immunity were prone to produce DSA after allogeneic therapy [28].

This discrepancy in the average cytotoxic scores over prior reports may be the result of different route of administration, since previous studies used the intradermal [14] whereas we assessed IA injection [16]. Because of the role of the skin as immune organ rich in blood and lymphatic vessels [29], stronger immune responses might be expected after intradermic MSC injection, whereas weak allo-antibody production was also reported in humans receiving IA allogeneic MSCs for knee OA [26]. The assessment of cytotoxic antibodies locally in the SF could have provided further information with this regard, but the limited availability of SF samples prevented its evaluation. Nevertheless, to the best of our knowledge, there are no reports on the local assessment of allo-antibodies after MSC administration.

Whereas recipients of MSC-primed, both mismatched and halfmatched, tended to produce fewer antibodies in vivo, these cells tended to be more targeted in the microcytotoxicity assays in vitro. These two observations, apparently opposed, might be due to a lower humoral response produced by MSC-primed after first injection because of their potentiated immune regulatory status [17]. However, after second injection, already formed antibodies would target these cells more easily because of their increased MHC expression.

Even though it is complex to correlate data from humoral response with clinical observations in the original in vivo study, it is worth noting that MSC-primed showed more capacity in vivo to regulate the inflammatory and catabolic joint environment at the short term [16], which might be related with higher capacity to evade the immune response thus producing fewer antibodies after first administration as suggested by this study. However, in the in vivo study, slight and transient local inflammation was noticed after second injection of MSC-primed, showing this group significant increase in the carpal perimeter and non-significant increase in the local temperature 24 h after re-injection and significant increase in synovial total white cell count 1 week after re-exposure that were not detected in the MSC-naïve group [16]. These clinical findings might be associated with targeting of MSC-primed by the antibodies produced after first injection, based on increased cell death observed when MSC-primed were used as target cells in microcytotoxicity assays in the present study. Therefore, even though the humoral response could be considered as mild based on cytotoxic scores, the antibody production could have a clinical impact by compromising the repeated administration of allogeneic cells, especially the primed ones.

## Conclusions

On the one hand, repeated IA injection of allogeneic MSCs (unstimulated) in mismatched horses resulted in both primary and secondary humoral responses. On the other hand, the immunomodulatory profile of MSCs primed with proinflammatory cytokines might have helped them to more effectively regulate the inflammation and to elicit lower primary humoral response when administered for the first time. However, if repeatedly injected, MSC-primed might be more easily targeted by pre-existing antibodies because of their increased expression of MHC. Nevertheless, partial compatibility between donor and recipient may help to avoid this secondary humoral response after re-exposure to MSC-primed. The immune response elicited against equine allogeneic MSCs and the factors influencing it must be further studied to develop more effective and safer cell therapies.

## Supplementary information

Additional file 1: Details of typing of equine leukocyte antigen (ELA) by intra-MHC microsatellites

Additional file 2: Evolution of cytotoxic scores along the time comparing different groups of recipients

## Data Availability

The datasets used and/or analyzed during the current study are available from the corresponding author on reasonable request.

## Abbreviations

APC: Allophycocyanin
BM: Bone marrow
DMEM: Dulbecco’s modified Eagle’s medium
DNA: Deoxyribonucleic acid
DSA: Donor-specific antibodies
EDTA: Ethylenediaminetetraacetic acid
ELA: Equine leukocyte antigen
FACS: Fluorescence-activated cell sorter
FBS: Fetal bovine serum
FITC: Fluorescein-5-isothiocyanate
HLA: Human leukocyte antigen
IA: Intraarticular
IFNγ: Interferon gamma
MHC: Major histocompatibility complex
MSCs: Mesenchymal stem cells
OA: Osteoarthritis
PBL: Peripheral blood lymphocyte
PBS: Phosphate buffered saline
PCR: Polymerase chain reaction
RT: Room temperature
SF: Synovial fluid
T: Time
TNFα: Tumor necrosis factor alpha
