# Supplementary material for: Allo-antibody production after intraarticular administration of mesenchymal stem cells (MSCs) in an equine osteoarthritis model: effect of repeated administration, MSC inflammatory stimulation, and equine leukocyte antigen (ELA) compatibility
Source: Stem Cell Res Ther. 2020 Feb 7;11:52. doi: 10.1186/s13287-020-1571-8 (PMC7006079; doi:10.1186/s13287-020-1571-8)
Supplement: Supplementary file 1 — Details of typing of equine leukocyte antigen (ELA) by intra-MHC microsatellites [file 13287_2020_1571_MOESM1_ESM.pdf]

Additional file 1. Typing of equine leukocyte antigen (ELA) by intra-MHC microsatellites.

Haplotypes found in 17 of the 18 animals assessed in this study are presented in this table together with their counts. Since there were no homozygotes, neither family relation was known, haplotypes were assigned just provisionally. Arrangement of genotypes into haplotypes started with two of the animals that were homozygotes for all the MHC-II microsatellite markers and then we looked for this pattern in other animals to also match with MHC-I and III markers. Thus, we found the same combination of the 10 alleles in 5 animals and provisionally assigned the first haplotype (ZAR01). Once one haplotype was arranged, other haplotypes could be provisionally assembled by observation. New provisional haplotypes were named by the prefix ZAR (from Universidad de Zaragoza) and a number, also considering the following:

* Provisional haplotypes that could be assembled because of high similarity with other observed haplotypes were named using the same number plus a letter. Italics are used to remark the allele/s that was/were different.

** Provisional haplotypes ZAR21 and ZAR31 were named as a mix of ZAR02/ZAR01 and ZAR03/ZAR01 haplotypes, respectively, because of the apparent recombination of other frequent haplotypes. Direct recombination events have been observed in previous studies and two hemi-haplotypes system has been proposed (Sadeghi et al., 2017; Holmes et al., 2019). For ZAR21 haplotype, observe that alleles for MHC class I and III are shared with ZAR02, whereas alleles for MHC class II are the same than in ZAR01a and ZAR01b. For ZAR31, alleles for MHC class I and III, and for the first two MHC class II markers, are the same than for ZAR03a haplotype, and the remaining class II alleles are shared with ZAR01 and ZAR01a haplotypes.

New alleles are indicated in bold: one allele for the marker ABGe9030 (205-6) and one allele for COR112 (270) that were not previously described (Sadeghi et al., 2017; Holmes et al., 2019) were observed in these animals. In particular, allele 205-6 received this name because it was observed just in the middle between alleles 205 and 206, previously described and available in reference samples kindly provided by Dr Antzack and Dr Miller. After checking with the Antczak laboratory, it seems that this new allele would be 205 whereas previous 205 maybe should be revisited as 204. In order to follow the nomenclature already established, we decided to maintain the 205-6 by now.

	INTRA-MHC MICROSATELLITES										
	MHC-I		MHC-III		MHC-II						
Haplotype	UMNJH-38	COR110	ABGe9019	UMNe65	ABGe9030	EQMHC1	COR112	COR113	UM011	COR114	Counts
ZAR01	156	207	301	263	212	192	254	260	172	243	**5**
ZAR01a*	156	*223*	301	263	212	192	254	260	172	243	**1**
ZAR01b*	156	207	301	263	212	192	254	260	172	*245*	**1**
ZAR02	165	211	303	259	**205-6**	192	260	268	167	247	**3**
ZAR21**	165	211	303	259	212	192	254	260	172	243	**1**
ZAR03	165	211	314	257	**205-6**	192	248	270	184	245	**2**
ZAR03a*	165	211	314	257	*210*	*190*	248	270	184	245	**1**
ZAR31**	165	211	314	257	210	190	254	260	172	243	**1**
ZAR04	165	215	303	259	212	190	**270**	266	170	245	**1**
ZAR05	156	197	303	259	210	190	254	274	169	247	**1**
ZAR06	156	219	314	257	**205-6**	192	268	278	175	241	**2**
ZAR07	156	197	314	261	211	194	264	266	169	251	**3**
ZAR07a*	156	*209*	*301*	*259*	211	194	264	266	169	251	**1**
ZAR08	156	207	303	259	219	190	**270**	278	175	241	**2**
ZAR09	156	215	301	257	219	190	254	260	172	243	**2**
ZAR10	156	221	312	261	211	192	262	268	176	247	**1**
ZAR11	156	197	314	261	**205-6**	192	264	260	179	251	**1**
ZAR12	163	197	303	259	210	190	254	260	172	243	**1**
ZAR13	156	207	314	261	219	196	264	270	172	249	**1**
ZAR14	156	215	307	257	206	192	256	270	174	234	**1**
ZAR15	156	207	314	259	215	194	258	270	172	249	**1**
ZAR16	163	197	303	259	**205-6**	192	248	270	184	245	**1**
