# Supplementary material for: Allo-antibody production after intraarticular administration of mesenchymal stem cells (MSCs) in an equine osteoarthritis model: effect of repeated administration, MSC inflammatory stimulation, and equine leukocyte antigen (ELA) compatibility
Source: Stem Cell Res Ther. 2020 Feb 7;11:52. doi: 10.1186/s13287-020-1571-8 (PMC7006079; doi:10.1186/s13287-020-1571-8)
Supplement: Supplementary file 2 — Evolution of cytotoxic scores along the time comparing different groups of recipients [file 13287_2020_1571_MOESM2_ESM.pdf]

Additional file 2.- Evolution of cytotoxic scores along the time comparing different groups of recipients

Cytotoxic scores among the three recipients groups (naïve-mismatched, primed-halfmatched and primed-mismatched) were not statistically different except for some specific situations in which primed recipients (both halfmatched and mismatched) presented lower scores than naïve recipients. Specifically, 1:16 diluted sera from both mismatched and halfmatched MSC-primed recipients presented significantly ($p<0.05$) lower scores than sera from naïve-mismatched recipients at T1 when assayed against PBLs (panel G). At T2, 1:2 diluted sera from primed-halfmatched (panel F) and 1:16 diluted sera from primed-mismatched (panel I) recipients also showed significantly lower scores ($p<0.05$) than sera from naïve recipients when assayed with MSC-primed as target cells. Finally, 1:16 diluted sera from primed-halfmatched ($p<0.01$) and from primed-mismatched ($p<0.05$) showed lower scores than sera from naïve-recipients at T4 when tested against MSC-primed.

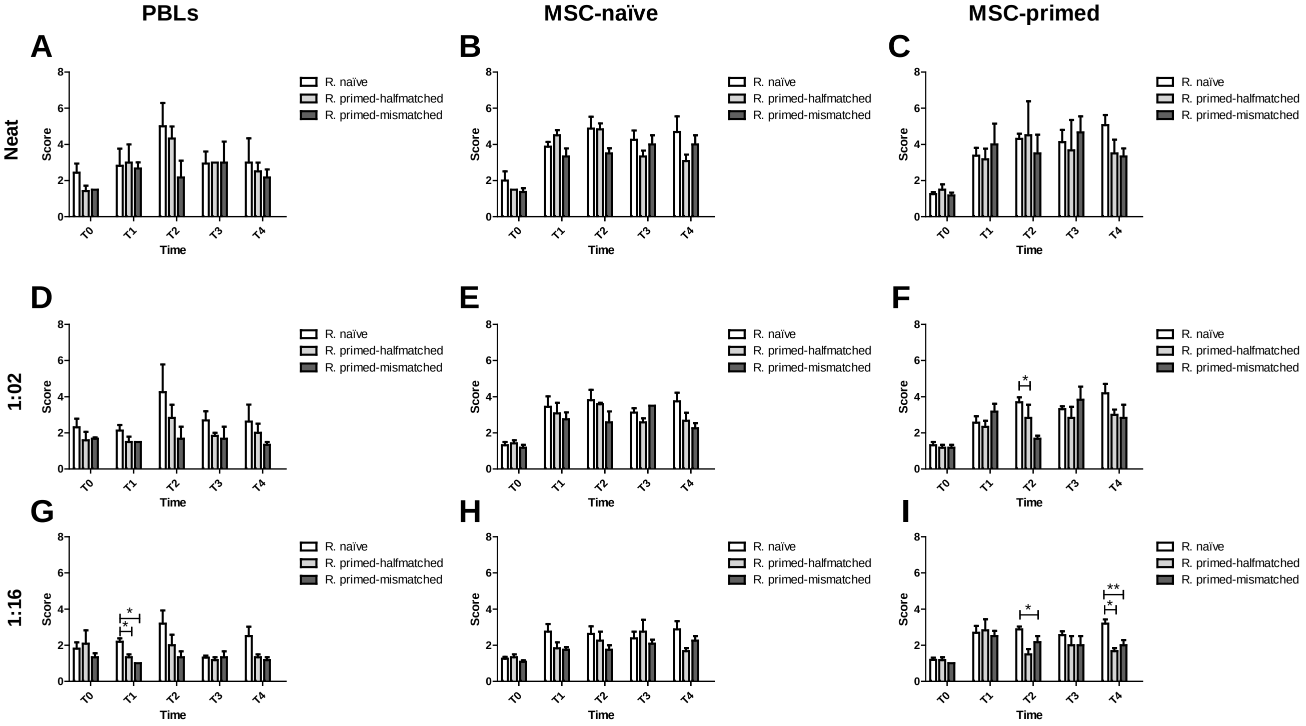

Mean ± S.E.M. of cytotoxic scores (Y axis) assigned to neat sera (top row; A, B, C), 1:2 diluted sera (middle row; D, E, F) and 1:16 diluted sera (bottom row; G, H, I) from recipients of MSC-naïve (white bars) and MSC-primed, halfmatched (light grey bars) and mismatched (dark grey bars), along the time (X axis; T0, pre-administration of corresponding MSCs; T1, one week after first MSC administration; T2, three weeks after first MSC administration- just before the second MSC administration; T3, one week after second MSC administration; T4, 90 days after second MSC administration), when assayed against different target cells: PBLs, peripheral blood lymphocytes (left column; A, D, G); MSC-naïve, unstimulated mesenchymal stem cells (middle column; B, E, H); MSC-primed, mesenchymal stem cells stimulated with tumor necrosis factor alpha and interferon before administration (right column, C, F, I). Asterisks (*) point out statistically significant differences (*=$p<0.05$, **=$p<0.01$) between recipient groups at one particular time-point.
